# Abdominal Wall Sinus: A Late Complication of Gallstone Spillage During Laparoscopic Cholecystectomy

**DOI:** 10.1155/1997/57313

**Published:** 1997

**Authors:** Michael D. Graham, Paul G. Anderson, James Toouli

**Affiliations:** Gastrointestinal Surgical Unit Flinders Medical Centre Australia

## Abstract

Long term complications of laparoscopic cholecystectomy are uncommon. However, as experience with this procedure accumulates, sporadic reports of non-biliary complication have been published. We report a case of abdominal wall sinus formation secondary to gallbladder perforation and stone spillage occurring during laparoscopic cholecystectomy.


*HPB Surgery*, 1997, Vol. 10, pp.163–164
Reprints available directly from the publisher
Photocopying permitted by license only

© 1997 OPA (Overseas Publishers Association)
Amsterdam B.V. Published in The Netherlands
by Harwood Academic Publishers
Printed in India

### CASE REPORT Abdominal Wall Sinus: A Late Complication of Gallstone Spillage During Laparoscopic Cholecystectomy

MICHAEL D GRAHAM, PAUL G ANDERSON and JAMES TOOULI

Gastrointestinal Surgical Unit Flinders Medical Centre

*(Received 28 November 1995)*

Long term complications of laparoscopic cholecystectomy are uncommon. However, as experience with this procedure accumulates, sporadic reports of non-biliary complication have been published. We report a case of abdominal wall sinus formation secondary to gallbladder perforation and stone spillage occurring during laparoscopic cholecystectomy.

KEY WORDS: Laparascopic cholecystectomy gallbladder perforation sinus

### MATERIALS AND METHODS

BF, a 70 year old female was admitted to the Flinders Medical Centre with a two day history of RUQ pain. She was febrile (38.9°) with a pulse of 100 beats/min. and tender in the right upper quadrant. Her WBC was $8.1 \times 10^3$ (cells/mm$^3$), alkaline phosphatase 158 (30–120 U/1), alanine transferase 196 (<40 U/1) and bilirubin 52 (<20 umol/1). Biliary tract ultrasound confirmed the presence of multiple gall stones in a thick-walled gallbladder. The common bile duct was of normal calibre. She was commenced on intravenous fluids, analgesia and broad spectrum anti-biotic therapy and underwent laparoscopic cholecystectomy 48 hours after admission.

At operation the gallbladder was distended with a friable oedematous wall and contained multiple stones. Operative cholangiography did not reveal any stones in the biliary tree. During the dissection a hole was made in the gallbladder fundus with grasping forceps and accidental spillage of a number of stones into the abdominal cavity occured. Not all the stones were retrieved. The gallbladder was extracted through the umbilical port. Post-operative recovery was uneventful and she left hospital two days later. She was reviewed two weeks later and noted to be totally asymptomatic. No further follow up was planned.

The patient represented five months later with intermittent discharge of purulent material from the right loin and the umbilical incision. The discharge had a scanty growth of *Klebsiella oxytoca*. Ultrasound demonstrated a collection in the right loin which was drained percutaneously. Three weeks later a sinogram demonstrated that a minimal residual cavity existed and the drain was removed. The discharge however persisted from the right loin and a further sinogram demonstrated a connection with a 7 × 6 × 5 cm cavity.

The sinus was explored under a general anaesthetic and was noted to be retroperitoneal. It branched with a superior limb ending blindly and an inferior limb ending in a large cavity in the right loin. The superior limb contained three gallstones (10,2,2 mm) at its apex. The tracts were curetted and layed open and allowed to heal by granulation. The umbilical port site was also explored and found to contain one small stone (2mm) with transverse colon firmly adherent to its posterior aspect. No direct communication could be demonstrated with the colon which was dissected free, the linea alba closed and the skin sutured. Both

*Correspondence to*: Professor James Toouli, Gastrointestinal Surgical Unit, Department of Surgery, Flinders Medical Centre, Bedford Park, South Australia 5042.

wounds healed uneventfully and there has been no recurrence of symptoms after six months.

### DISCUSSION

Since the introduction of laparoscopic cholecystectomy into clinical practice, it has now become the procedure of choice for dealing with symptomatic gallstones. There have been several large series detailing both intra-operative and early post-operative complications [1,2], however little is known about the long term morbidity.

Gallstone spillage occurs in up to 20% of cases[3], usually, as a result of gallbladder perforation occurring during either the initial dissection or during extraction through the umbilical or epigastric trocar insertion sites. In one published series, gallstone spillage was considered as an indication for conversion to an open procedure[4] to ensure complete retrieval of all stones, however a more common practice is to remove as many spilled stones as possible laparoscopically followed by irrigation of the abdominal cavity by normal saline.

The fate of spilled stones is unknown. Clinical and experimental studies have shown that intraperitoneal abscess formation may occur around gallstones. These may discharge through surgical scars[5], especially trocar insertion sites. Stones may eventually migrate into the bronchial tree and be coughed up[6]. It appears likely that spilled gallstones form a nidus of infection with resulting abscess formation which later discharges spontaneously via adjacent structures. This case of abdominal wall sinus formation following gallstone spillage at laparoscopic cholecystectomy highlights the importance, where possible, of retrieving spilled gallstones from the abdominal cavity.

To reduce the likelihood of spilling gallstones during the procedure, we suggest that excessive traction on the gallbladder be avoided during the initial dissection. Repeated "handling" of the gallbladder should be done with non-toothed forceps, the gallbladder contents aspirated and any large stones removed using Desjardins forceps prior to extraction under vision through the umbilical incision which should readily be enlarged to acommodate the gallbladder. In the event of a perforation occurring, this should immediately be sealed using an Endoloop. If the hole has not been sealed successfully, the gallbladder should be placed in a plastic bag for extraction. Spilled stones should be retrieved where possible and copious irrigation used to remove bile. We do not recommend conversion to laparotomy to retrieve spilled stones.

### REFERENCES

1. The Southern Surgeons Club: (1991) A prospective analysis of 1518 laparoscopic cholecystectomies. *N. Eng. J. Med.*, **324** (16), 1073–1078.
2. The European Experience with Laparoscopic Cholecystectomy. Cuschieri, A., Dubois, F., Mouiel, J., Mouret, P., Becker, H., Buess, G., Trede, M & Troidl, H. (1991) *Am. J. Surg.*, **161**, 385–387.
3. Gallbladder and gallstone removal, open versus closed laparotomy, and pneumoperitoneum. Fitzgibbons, R.J., Annibali, R. & Litke BS. (1993) *Am. J. Surg.*, **165**, 497–504.
4. Complications of laparoscopic cholecystectomy. (1991) Ponsky. JL. *Am. J. Surg.*, **161**, 393–395.
5. Lost Intraperitoneal Stones After Laparoscopic Cholecystectomy: Harmless Sequela or Reason for Reoperation? Catarci M, Zaraca F, Scaccia. M., & Carboni. M., (1994) *Surgical Laparoscopy and Endoscopy.*, **3**, **4**, 318–322.
6. Cholelithoptysis and Cholelithorrhea: Rare Complications of Laparoscopic Cholecystectomy. Lee, V.S., Paulson, E.L., Flannery, J.E. & Meyers, W.C. (1993) *Gastroenterology.*, **105**, 1877–1881.

### COMMENTARY FOR MANUSCRIPTS #261 AND #281

These two papers make a very important point - clean up after your laparoscopic cholecystectomies! I am guilty! As author of the Southern Surgeons' Club original report, I suggested that spilled stones might be innocuous. I never said this, but the data that showed that spilled stones led to no complications spoke for themselves. This was a short term study! Our paper in Gastroenterology (Lee, *et.al.*, 1993, Gastroenterology 105:1877–1881. Cholelithoptisis and cholelithorrhea after laparoscopic cholecystectomy on hull) made this point graphically. Now, more reports such as these are occuring! Spilled stones after laparoscopic cholecystectomy on hull can cause problems months or years after surgery! All you surgeons out there: clean up after your laparoscopic cholecystectomy on hull even if you have to convert!

William C. Meyers, M.D.
Professor and Chief of Surgery
University of Massachusetts

